# White Matter Hyperintensity Volume and Poststroke Cognition: An Individual Patient Data Pooled Analysis of 9 Ischemic Stroke Cohort Studies

**DOI:** 10.1161/STROKEAHA.123.044297

**Published:** 2023-10-30

**Authors:** Floor A.S. de Kort, Mirthe Coenen, Nick A. Weaver, Hugo J. Kuijf, Hugo P. Aben, Hee-Joon Bae, Régis Bordet, Guido Cammà, Christopher P.L.H. Chen, Anna Dewenter, Marco Duering, Rong Fang, Ruben S. van der Giessen, Olivia K.L. Hamilton, Saima Hilal, Irene M.C. Huenges Wajer, Cheuk Ni Kan, Jonguk Kim, Beom Joon Kim, Sebastian Köhler, Paul L.M. de Kort, Peter J. Koudstaal, Jae-Sung Lim, Renaud Lopes, Vincent C.T. Mok, Julie Staals, Narayanaswamy Venketasubramanian, Charlotte M. Verhagen, Frans R.J. Verhey, Joanna M. Wardlaw, Xin Xu, Kyung-Ho Yu, J. Matthijs Biesbroek, Geert Jan Biessels

**Affiliations:** 1 Department of Neurology and Neurosurgery, UMC Utrecht Brain Center, the Netherlands (F.A.S.d.K., M.C., N.A.W., G.C., I.M.C.H.W., C.M.V., J.M.B., G.J.B.).; 2 Image Sciences Institute, University Medical Center Utrecht, the Netherlands (H.J.K.).; 3 Department of Neurology, Elisabeth Tweesteden Hospital, Tilburg, the Netherlands (H.P.A., P.L.M.d.K.).; 4 Department of Neurology, Seoul National University Bundang Hospital, Seoul National University College of Medicine, Seongnam, Republic of Korea (H.-J.B., J.K., B.J.K.).; 5 Lille Neuroscience & Cognition (LilNCog) U1172, Université Lille, Inserm, CHU Lille, France (R.B., R.L.).; 6 Department of Pharmacology, Yong Loo Lin School of Medicine, National University of Singapore (C.P.L.H.C., S.H., C.N.K., X.X.).; 7 Memory, Aging and Cognition Center, National University Health System, Singapore (C.P.L.H.C., S.H., C.N.K., X.X.).; 8 Institute for Stroke and Dementia Research (ISD), University Hospital, LMU Munich, Germany (A.D., M.D., R.F.).; 9 Medical Image Analysis Center (MIAC) and Department of Biomedical Engineering, University of Basel, Switzerland (M.D.).; 10 Department of Neurology, Erasmus Medical Center, Rotterdam, the Netherlands (R.S.v.d.G., P.J.K.).; 11 Neuroimaging Sciences, Centre for Clinical Brain Sciences, University of Edinburgh, United Kingdom (O.K.L.H., J.M.W.).; 12 UK Dementia Research Institute at the University of Edinburgh, United Kingdom (O.K.L.H., J.M.W.).; 13 MRC/CSO Social and Public Health Sciences Unit, School of Health and Wellbeing, University of Glasgow, United Kingdom (O.K.L.H.).; 14 Saw Swee Hock School of Public Health, National University of Singapore and National University Health System (S.H.).; 15 Experimental Psychology, Helmholtz Institute, Utrecht University, the Netherlands (I.M.C.H.W.).; 16 Department of Psychiatry and Neuropsychology, School for Mental Health and Neuroscience, Maastricht University, the Netherlands (S.K., F.R.J.V.).; 17 Department of Neurology, Asan Medical Center, University of Ulsan College of Medicine, Seoul, Republic of Korea (J.-S.L.).; 18 Division of Neurology, Department of Medicine and Therapeutics (V.C.T.M.), The Chinese University of Hong Kong.; 19 Lau Tat-Chuen Research Centre of Brain Degenerative Diseases in Chinese, Li Ka Shing Institute of Health Sciences, Gerald Choa Neuroscience Institute, Lui Chi Woo Institute of Innovative Medicine (V.C.T.M.), The Chinese University of Hong Kong.; 20 Department of Neurology, Maastricht University Medical Center, the Netherlands (J.S.).; 21 Raffles Neuroscience Centre, Raffles Hospital, Singapore (N.V.).; 22 Department of Neurology, Hallym University Sacred Heart Hospital, Hallym University College of Medicine, Anyang, Republic of Korea (K.-H.Y.).; 23 Department of Neurology, Diakonessenhuis Hospital, Utrecht, the Netherlands (J.M.B.).

**Keywords:** brain, cerebral small vessel diseases, cognition, infarcts, ischemic stroke, neuroimaging

## Abstract

**BACKGROUND:**

White matter hyperintensities (WMH) are associated with cognitive dysfunction after ischemic stroke. Yet, uncertainty remains about affected domains, the role of other preexisting brain injury, and infarct types in the relation between WMH burden and poststroke cognition. We aimed to disentangle these factors in a large sample of patients with ischemic stroke from different cohorts.

**METHODS:**

We pooled and harmonized individual patient data (n=1568) from 9 cohorts, through the Meta VCI Map consortium (www.metavcimap.org). Included cohorts comprised patients with available magnetic resonance imaging and multidomain cognitive assessment <15 months poststroke. In this individual patient data meta-analysis, linear mixed models were used to determine the association between WMH volume and domain-specific cognitive functioning (*Z* scores; attention and executive functioning, processing speed, language and verbal memory) for the total sample and stratified by infarct type. Preexisting brain injury was accounted for in the multivariable models and all analyses were corrected for the study site as a random effect.

**RESULTS:**

In the total sample (67 years [SD, 11.5], 40% female), we found a dose-dependent inverse relationship between WMH volume and poststroke cognitive functioning across all 4 cognitive domains (coefficients ranging from −0.09 [SE, 0.04, *P*=0.01] for verbal memory to −0.19 [SE, 0.03, *P*<0.001] for attention and executive functioning). This relation was independent of acute infarct volume and the presence of lacunes and old infarcts. In stratified analyses, the relation between WMH volume and domain-specific functioning was also largely independent of infarct type.

**CONCLUSIONS:**

In patients with ischemic stroke, increasing WMH volume is independently associated with worse cognitive functioning across all major domains, regardless of old ischemic lesions and infarct type.

Poststroke cognitive impairment (PSCI) is a major cause of long-term morbidity and mortality and occurs in about half of patients with ischemic stroke.^1–3^ PSCI occurrence is likely determined by features of the acute infarct, such as infarct location and size, against the background of preexisting brain injury and other patient-related factors, such as age and educational level.^4^ In this context, cerebral small vessel disease (cSVD) is of particular interest as white matter hyperintensities (WMH), a key manifestation of cSVD, have been linked to PSCI risk (systematic review^5^).

The relationship between WMH and PSCI may be influenced by multiple factors, including infarct type. For example, the burden of WMH is known to be larger in patients with recent small subcortical infarcts compared with those with large thrombo-embolic infarcts.^6^ Yet, patients with large infarcts are more likely to develop PSCI than those with small subcortical infarcts.^1,7^ This interplay between infarct type, WMH burden, and PSCI needs further evaluation, also considering other common preexisting brain injury, in particular lacunes, old infarcts, and brain atrophy.^8,9^

PSCI is a complex construct and can involve multiple cognitive domains, with substantial interindividual variation.^1,7^ Conventionally, WMH are often primarily linked to deficits in executive functioning and processing speed (PS). Yet, such domain specificity for WMH has recently been questioned (systematic review^10^) and has not been sufficiently studied after ischemic stroke. Furthermore, PSCI is mostly operationalized in a dichotomous fashion, in terms of presence or absence, whereas cognitive functioning after stroke clearly is a continuum.

In this study, we aimed to determine the relation between WMH volume and domain-specific cognitive functioning, also considering infarct type and other preexisting brain injury, in a large sample of patients with ischemic stroke from different cohorts.

## METHODS

The data that support the findings of this study are available from the corresponding author/project leads on reasonable request. Restrictions related to privacy and personal data sharing regulations and informed consent may apply.

### Patient Selection

**Figure 1.**
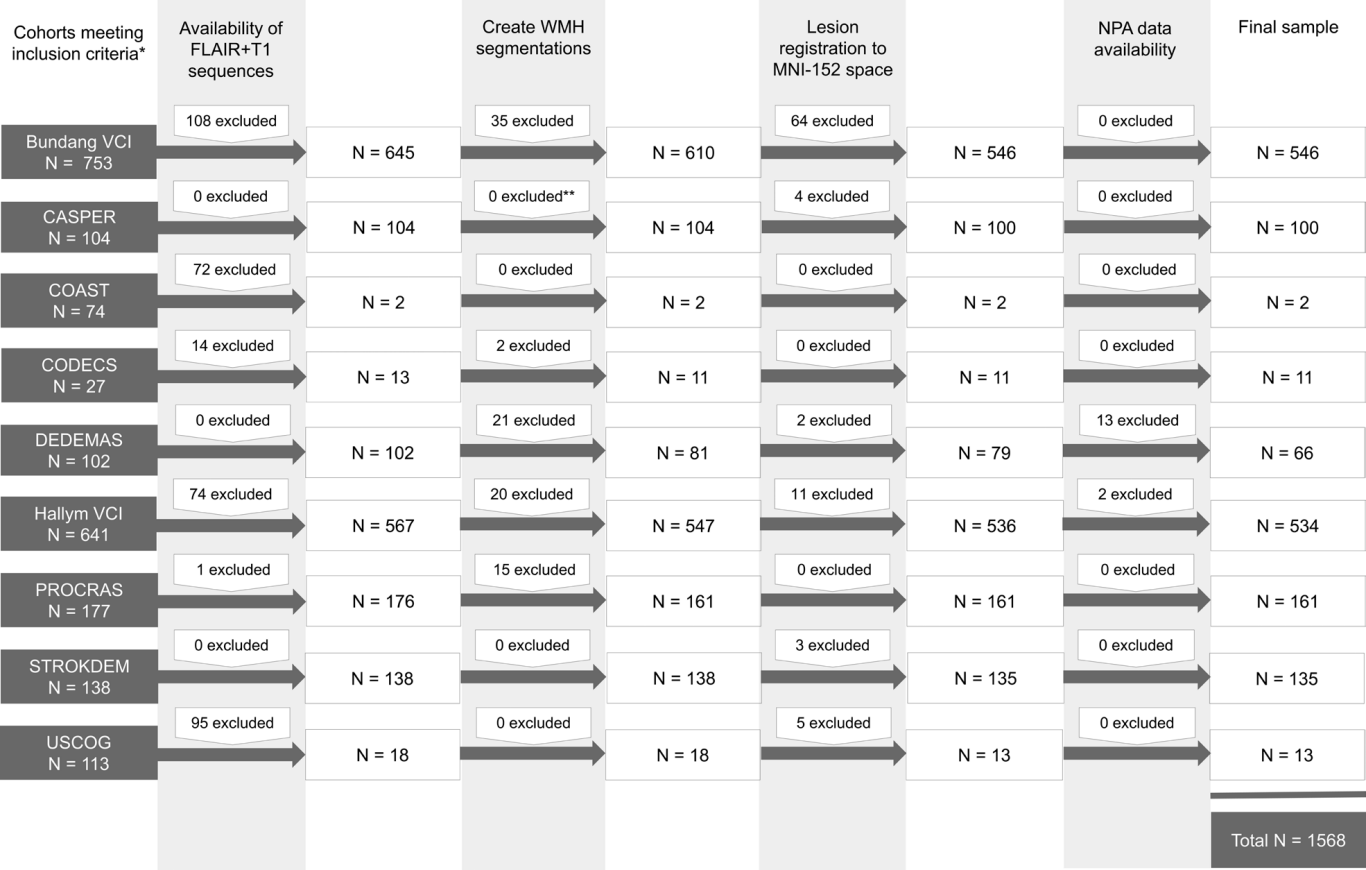
**Flowchart of patient selection.** *Cohorts previously participating in the pilot study^11^ or study on strategic infarct locations^7^ and with available domain-specific cognitive testing. **White matter hyperintensities (WMH) segmentations performed by cohort. CASPER indicates Cognition and Affect after Stroke: A Prospective Evaluation of Risks; COAST, Cognitive Outcome After Stroke; CODECS, Cognitive Deficits in Cerebellar Stroke; DEDEMAS, Determinants of Dementia After Stroke; NPA, neuropsychological assessment; PROCRAS, Prediction of Cognitive Recovery After Stroke; STROKDEM, Study of Factors Influencing Post-Stroke Dementia; USCOG, Utrecht Stroke and Cognition; and VCI, Vascular Cognitive Impairment.

We pooled and harmonized individual patient data from 9 ischemic stroke cohorts: France (STROKDEM [Study of Factors Influencing Post-Stroke Dementia]), Germany (DEDEMAS [Determinants of Dementia After Stroke]), the Netherlands (CASPER [Cognition and Affect after Stroke: A Prospective Evaluation of Risks], CODECS [Cognitive Deficits in Cerebellar Stroke], PROCRAS [Prediction of Cognitive Recovery After Stroke], USCOG [Utrecht Stroke and Cognition]), Singapore (COAST [Cognitive Outcome After Stroke]), and South Korea (Bundang VCI [Vascular Cognitive Impairment], Hallym VCI; cohort details in Supplemental Material). Eligible cohorts were derived from either the Meta VCI Map consortium PSCI pilot study^11^ or strategic infarct location study.^7^ For the current study, 9 cohorts with domain-specific neuropsychological assessment within 15 months of the index stroke were included. Individual patients were selected from these cohorts according to the availability of the following: (1) acute infarct segmentations in Montreal Neurological Institute (MNI) space, (2) magnetic resonance imaging (MRI) with fluid-attenuated inversion recovery and T1 sequences and (3) cognitive test results on preselected domain-specific tests. Note that in some cohorts, few patients remained due to the limited availability of MRI. The flowchart of the final patient selection is shown in Figure 1. Central data processing and analysis were done at the University Medical Center Utrecht (Utrecht, the Netherlands). For all cohorts, ethical and institutional approval was obtained as required by local regulations to allow data acquisition, including informed consent, and data sharing. Background and organization of the Meta VCI Map consortium are described in a design article^11^ and on the consortium website www.metavcimap.org.

### Harmonization of Clinical Characteristics

Geographic region was dichotomized as Europe (the Netherlands, Germany, France) or Asia (South Korea, Singapore). Harmonization of educational level was done as described previously, by recoding the original education data into a 4-category variable according to the approach in the Stroke and Cognition consortium.^7^

### Cognitive Data Harmonization

All 9 cohorts provided individual norm-referenced neuropsychological test scores. Tests for the pooled analyses were selected based on availability between cohorts and were assigned to 4 cognitive domains: (1) attention and executive functioning (AEF); (2) PS; (3) language; and (4) verbal memory (VM). Assignment of tests to specific cognitive domains was based on prior work.^7^ Mean cognitive domain *Z* scores were created (see Supplemental Material for details), where, for example, a mean *Z* score of −1 implies that patients perform on average 1 SD (ie, at the 16th percentile) below the normative mean.

### Image Processing

Details on image processing steps and visual ratings are described in the Supplemental Material. In short, WMH segmentations were performed in Utrecht for 8 cohorts^12^ and provided by the participating center for 1 cohort (CASPER).^13^ WMH maps were registered to the 1×1×1 mm resolution MNI-152 brain template^14^ for spatial normalization, using RegLSM.^15^ An expert (M.C.) with extensive experience in WMH segmentations visually inspected all segmentations and registrations. Failed segmentations (n=93, 5.3%) and registrations (n=89, 5.3%) were excluded. Acute infarct segmentations in MNI space, available from prior Meta VCI Map projects,^7,11^ were subtracted from the derived WMH maps. Normalized volumes for the acute infarct and WMH were calculated on the MNI-152 template. Figure S1 shows 3 examples of WMH lesion maps and corresponding acute infarct lesion maps in the MNI-152 template. The large sample size enabled stratified analyses for different acute infarct types, defined as the following: (1) small subcortical infarcts (supratentorial infarcts with a lesion volume of ≤4.19 mL, compatible with diameter ≤2 cm, following Standards for Reporting Vascular Changes on Neuroimaging criteria^16^); (2) larger supratentorial infarcts with or without cortical involvement, henceforth, referred to as large infarcts; and (3) infratentorial infarcts (any infarct involving brainstem or cerebellum regardless of size). Patients with acute infarcts in both supratentorial and infratentorial regions were included in both subgroup analyses (n=69). Lacunes and old infarcts were rated visually by 2 independent raters (F.K. and J.M.B. or F.K. and G.J.B) and processed centrally for all but 1 cohort (CASPER). Because intracranial volume/whole brain segmentations failed in a substantial number of patients, brain parenchymal fraction calculations, as a substitute for atrophy, were only available for 27% of participants.

### Statistical Analyses

In this individual patient data meta-analysis, linear mixed models were used to assess the independent effect of WMH volume on cognitive domain *Z* scores across the 4 tested domains in the total dataset. First, univariate models were used with WMH volume as the independent variable (log10-transformed, fixed effect) and cognitive domain *Z* scores as the dependent variable. Subsequently, multivariable models were used to enable correction for possible confounders. Covariates were selected based on literature rather than significant relations with cognition and included clinical variables: age, sex, educational level, geographic region, and imaging parameters: acute infarct volume (log10-transformed), presence of old infarcts, and presence of lacunes. Categorical variables were treated as dummy variables. Covariates were checked for colinearity and were entered into the model as fixed effects. All univariate and multivariate analyses were corrected for the study site as a random effect. A *P* value of <0.05 was considered statistically significant. To determine whether the obtained results were affected by infarct type, we performed stratified analyses for large, small subcortical, and infratentorial infarcts. Finally, we performed supplementary analyses taking into account the location of the acute infarct (by stratifying for the location impact score^7^) and atrophy (see Supplemental Material for details). All analyses were performed using glmnet (v4.1.3) and lme4 (v1.1.26) in R (v4.1.2), https://cran.r-project.org/. This article follows the PRISMA (Preferred Reporting Items for Systematic Reviews and Meta-Analyses) Individual Patient Data statement.^17^

## RESULTS

### Study Population

We included 1568 patients from 9 cohorts (Figure 1). The mean age was 67.3 years (SD, 11.5), 626 (39.9%) were women, 69% of patients were Asian, and others were European. On MRI, old infarcts were present in 19.3% of patients and ≥1 lacunes in 36.5%. Mean norm-referenced *Z* scores on all cognitive domains were negative, with VM being the most affected domain (mean *Z* score, −0.95; Table 1). There was heterogeneity in patient characteristics between cohorts, reflecting differences in inclusion and exclusion criteria. The median normalized WMH volume was 7.1 mL and increased exponentially across WMH deciles (Table S3). Patients in the upper deciles of WMH volume were more likely to be women and older. Proportions of patients with hypertension, and diabetes also increased across deciles. Smoking (past or present) was less common in the upper deciles and this effect could not be attributed to sex (stratified analysis, data not shown). Patients in the upper deciles more often had a medical history of stroke or transient ischemic attack as well as old infarcts and in particular, lacunes on MRI. With regards to acute infarcts, patients in the upper deciles had higher scores on the National Institutes of Health Stroke Scale and were more likely to have a recent small subcortical infarct on MRI (Table S3).

**Table 1.**
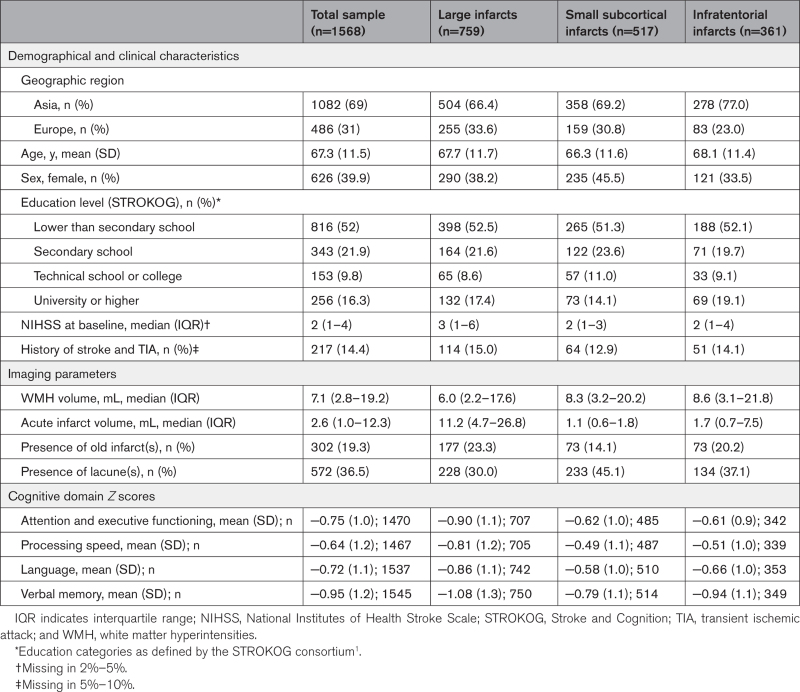
Baseline Characteristics

### Relation Between WMH Volume and Cognitive Functioning in the Total Sample

**Figure 2.**
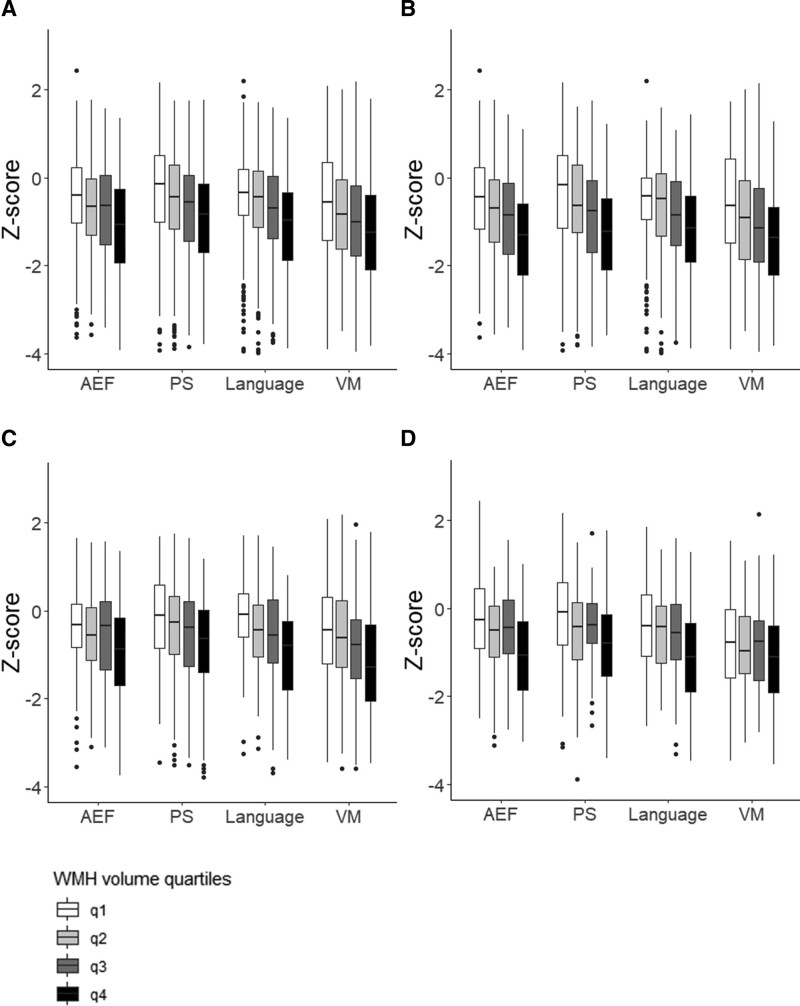
**Quartiles of unadjusted** white matter hyperintensities** (WMH) volume vs domain-specific cognitive functioning. A**, Total sample (n=1568), **B**, large infarcts (n=759), **C**, small subcortical infarcts (n=517), and **D**, infratentorial infarcts (n=361). Cognitive functioning is shown as mean *Z* score for each cognitive domain. AEF indicates attention and executive functioning; PS, processing speed; VM, verbal memory. All images were created using ggplot2 (v3.4.0) in R (v4.1.2).

There was a significant inverse relationship between WMH volume and poststroke cognitive functioning across all 4 tested domains. Effect estimates were lower for VM compared with the other domains (Table 2, univariate analysis). Figure 2A (total sample) shows the unadjusted relation between WMH volume (divided into quartiles for visualization), and domain-specific cognitive functioning (mean differences of cognitive *Z* scores between lowest and highest quartile: 0.64, 0.66, 0.67, and 0.57 for AEF, PS, language, and VM, respectively).

**Table 2.**
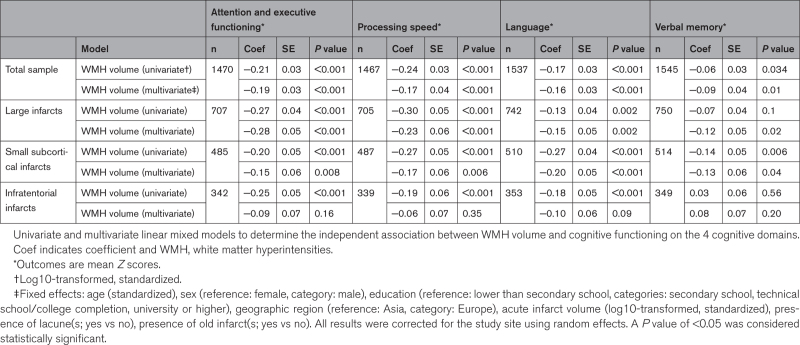
WMH Volume and Cognition

The relation between WMH volume and cognitive functioning across domains was independent of acute infarct volume, presence of old infarcts, and lacunes, age, sex, educational level, geographic region, and study site (Table 2, multivariate analysis). The random effect terms showed cohort-cohort variability, with coefficients ranging from 0.05 for AEF to 0.34 for VM. In sensitivity analyses in participants with available data on brain parenchymal fraction (n=422; 27%), effect sizes remained largely unchanged after adding brain parenchymal fraction to the model for the domains of AEF and PS. The influence of atrophy on the relation between WMH volume and the domains of language and VM could not be reliably assessed, details in Appendix S4.

### Results Stratified by Infarct-Subtype

Seven hundred fifty-nine patients had large infarcts, 517 patients had small subcortical, and 361 patients had infratentorial infarcts on MRI. Patients with large infarcts had higher acute infarct volumes, lower median WMH volumes, and lower mean cognitive *Z* scores compared with patients with small subcortical infarcts and infratentorial infarcts (details in Table 1).

In univariate analyses, effect sizes were mostly consistent with the overall analyses for each of the 3 infarct types, with the exception of the relation between WMH volume and VM performance which was not significant for infratentorial infarcts (Table 2). The unadjusted relation between WMH volume (divided into quartiles) and domain-specific functioning for each infarct type is also visualized in Figure 2.

For subgroups with large infarcts and small subcortical infarcts, results were independent of acute infarct volume, presence of old infarcts and lacunes, age, sex, educational level, and geographic region, consistent with the overall analyses, largely with similar effect sizes. For the subgroup with infratentorial infarcts, we did not find an independent relation between WMH volume and poststroke cognitive functioning after adjusting for confounders (multivariate analyses, Table 2).

In stratified analyses, according to the location of the acute infarct (using the location impact score^7^), the relation between WMH volume and cognition was strongest in those with the most strategic infarcts (details in Appendix S5).

## DISCUSSION

In this large-scale multicenter study of patients with ischemic stroke, we found a dose-dependent inverse relationship between WMH volume and poststroke cognitive functioning across all tested cognitive domains (AEF, PS, language, and VM). This relation was independent of acute infarct volume, presence of old infarcts and lacunes, and also largely independent of infarct type.

In contrast to the traditional view that WMH and other manifestations of cSVD primarily affect PS and AEF, we did not observe a specific cognitive profile associated with WMH volume, but rather found significant associations with all tested cognitive domains. Previous studies in patients with ischemic stroke often had limited sample size (ie, n<200),^18–21^ used visual rating scales to assess WMH burden^9,20–23^ and tested a limited number of cognitive domains.^18–20,23^ Across these studies, WMH burden was consistently found to be associated with deficits in AEF^9,18,19,21–23^ and PS.^9,18,21,22^ Associations with visuospatial functions^9,19,21–23^ and language^9,20^ have also been reported. About VM, 3 studies^19,22,23^ did report an association with the total burden of WMH, whereas 3 other studies, 1 of which included 648 patients with ischemic stroke, did not.^9,18,21^ Notably, the latter study did report an association between the number of lacunes and memory. In the current study, the effect estimate for the relation between WMH volume and VM was significant, albeit smaller, and with a higher cohort–cohort variability compared with the other domains. Yet, the overall picture of cross-domain cognitive deficits in relation to WMH burden that emerges is consistent with our results in, to our knowledge, the largest cohort of patients with ischemic stroke. Of note, comparable results of cross-domain cognitive deficits in relation to WMH volume were reported in a large memory-clinic-based study^24^ and this lack of domain specificity for cognitive deficits has also been noted in 2 large systematic reviews in patients with sporadic cSVD,^10^ and patients with vascular cognitive impairment not demented.^25^ Disruption of large-scale functional networks by WMH might underlie these cognitive deficits across domains.^26^

The association of WMH with cognitive outcomes was also robust when considering lacunes and old infarcts, despite these lesions being common in the included patients. We are not aware of any previous studies that adjusted for these prior injuries. Unfortunately, we could not obtain atrophy measures for all our participants, but the available results suggest a limited effect of global atrophy on the relation between WMH and cognitive outcomes, at least for the domains of AEF and PS. Previous research reported associations between markers of neurodegeneration itself, such as medial temporal lobe atrophy, and PSCI.^27–29^ One study showed an association between medial temporal lobe atrophy and WMH burden in patients with stroke.^30^ The possible interplay between WMH and markers of neurodegeneration in relation to cognition after ischemic stroke requires further exploration.^4,31^ The increasing availability of fluid and imaging-based markers of neurodegeneration may facilitate such efforts.

Effect estimates of the relation between WMH and cognition appeared to be largely consistent across infarct types. The cSVD burden, that is, WMH and lacunes, was relatively comparable between patients presenting with small subcortical and infratentorial infarcts, but lower in patients with large infarcts, in line with previous literature.^6,32^ Moreover, cognitive performance across domains was worse in patients with large infarcts than in patients with other infarct types, also consistent with prior observations.^1^ These interconnections between infarct type, cSVD, and cognitive outcomes underline the importance of our stratified analyses. The additional stratified analyses taking into account the strategic location of the acute infarct showed that effect estimates of the relation between WMH volume and cognition were highest for those with strategic infarcts. This may reflect a multihit effect, where the combination of 2 adverse factors synergistically affect the outcome, possibly by exhausting brain reserve.

Strengths of our study are the large sample size, which enabled stratification for infarct type, the availability of multidomain, norm-referenced cognitive assessment, and the uniform output of imaging data benefitting from central processing and rigorous quality controls. Furthermore, the volumetric quantification of WMH burden in combination with the continuous range of cognitive outcome scores enabled an in-depth analysis of the relation between the 2. Several potential limitations should also be noted. First, post hoc pooling of data from multiple cohorts inherently resulted in data heterogeneity. We, therefore, chose to select only cognitive tests that were available in multiple cohorts, but differences in timing may still have influenced our results. Nearly, all imaging data were processed centrally, with the use of previously published processing pipelines that can handle data from different scanners and sequences.^12^ Nevertheless, variability in the data would have been less if we could have used a single predefined scan protocol across centers. To account for differences between cohorts, we treated the study site as a random effect in all analyses. Second, some cohorts preselected patients with milder strokes. Moreover, extensive neurocognitive testing requires certain basic motor skills, language, and visual abilities that patients with severe stroke mostly lack, resulting in an overrepresentation of patients with mild to moderate stroke severity in our sample, also reflected by the average National Institutes of Health Stroke Scale on admission. Third, although patients with prestroke dementia were mostly excluded, that is, based on the Informant Questionnaire for Cognitive Decline in the Elderly score or prehospital diagnosis,^7^ prestroke cognitive functioning could have influenced our findings. Fourth, with the current selection criteria, some cohorts had few remaining patients. Finally, the subtraction of acute infarct maps from WMH maps might underestimate preexisting WMH volume, particularly in case of large infarcts. An alternative approach is to focus on WMH burden contralateral of the acute infarct,^20^ but this assumes symmetrical WMH distribution and cannot deal with bilateral infarcts. Nevertheless, our findings in the group with large infarcts were comparable to other infarct types suggesting no major impact of this subtraction effect.

Over the past years, there has been an extensive focus on the association between burden of WMH and poor outcomes after ischemic stroke, such as an increased risk of functional impairment,^33^ dementia, and mortality, as also illustrated in the systematic review and meta-analysis by Georgakis et al.^5^ On top of this evidence, the present study shows that increasing WMH volume is independently associated with worse cognitive functioning across all major domains, regardless of old ischemic lesions and infarct type. The diversity of our study population enhances the generalizability of our findings, at least to those with mild to moderately severe stroke. Our results underline the importance of preventive strategies and interventions that target cSVD progression in populations at risk for stroke. Furthermore, WMH volume can support personalized prediction of PSCI and may be considered when setting rehabilitation goals.^4^ Future research could focus on the improvement of current prediction models for PSCI, by adding WMH volume and possibly other vascular lesions to the model. Precise quantification and uniform definitions of imaging markers might facilitate such efforts.^9,16,31^

## ARTICLE INFORMATION

### Sources of Funding

The Meta VCI Map consortium is supported by Vici Grant 918.16.616 from ZonMW to Dr G.J. Biessels Harmonization analyses for this study were supported by a Rudolf Magnus Young Talent Fellowship from the University Medical Center Utrecht Brain Center to Dr J. Matthijs Biesbroek. Dr J.M. Wardlaw reports grants from the Row Fogo Charitable Trust, the Wellcome Trust, and the UK Dementia Research Institute which receives its funding from the UK Medical Research Council, Alzheimer’s Society and Alzheimer’s Research UK, during the conduct of the study; and grants from Fondation Leducq, EU Horizon 2020 (SVDs@target project, grant agreement number 666881), the British Heart Foundation, and the UK Stroke Association, outside the submitted work. Dr O.K.L. Hamilton is supported by the Medical Research Council [MC_UU_00022/2] and the Scottish Chief Scientist Office [SPHSU17]. Dr Aben reports grants from ZonMW. Dr C.P.L.H. Chen reports grants from the National Medical Research Council (NMRC) of Singapore and the National University of Singapore. Dr S. Köhler received governmental funding from the Netherlands Society of Science (NWO) and ZonMW. The CASPER study (Cognition and Affect After Stroke: A Prospective Evaluation of Risk) was supported by Maastricht University, Health Foundation Limburg, and Stichting Adriana van Rinsum-Ponsen. The PROCRAS cohort (Prediction of Cognitive Recovery After Stroke) was funded via ZonMW as part of the TopZorg project in 2015 (grant number 842003011). The CODECS cohort ([Cognitive Deficits in Cerebellar Stroke]; ongoing) is supported by a grant from Stichting Coolsingel (grant number 514). The DEDEMAS cohort (Determinants of Dementia After Stroke) was funded by the Vascular Dementia Research Foundation.

### Disclosures

Dr H.-J. Bae reports grants from Chong Gun Dang Pharmaceutical Corp and Korean Drug Co, Ltd outside of the submitted work. Dr G.J. Biessels reports grants from The Netherlands Organisation for Health Research and Development (ZonMW), during the conduct of the study. The other authors report no conflicts.

### Supplemental Material

Supplemental Methods

Supplemental Results

Figure S1

Tables S1–S3

Appendices S4–S5

References 34–39

## Supplementary Material
