# Supplementary material for: White Matter Hyperintensity Volume and Poststroke Cognition: An Individual Patient Data Pooled Analysis of 9 Ischemic Stroke Cohort Studies
Source: Stroke. 2023 Oct 30;54(12):3021–9. doi: 10.1161/STROKEAHA.123.044297 (PMC10664782; doi:10.1161/STROKEAHA.123.044297)
Supplement: Supplementary file 1 [file str-54-3021-s001.pdf]

Supplemental material

Supplemental methods

Image processing

Figure S1: Examples of white matter hyperintensity and acute infarct lesion maps on the MNI-152 template

Cognitive data processing and harmonization

Table S1: selection of cognitive tests per cohort

Data quality control procedures

Supplemental results

Table S2. Cohort-specific baseline characteristics

Table S3. Baseline characteristics stratified by decile of white matter hyperintensity volume

Appendix S4. Sensitivity analysis: brain parenchymal fraction

Appendix S5. Sensitivity analysis: location impact score

All references are listed in the main manuscript.

Supplemental methods

<u>Image processing</u>

WMH segmentations

For eight cohorts WMH segmentations were performed in Utrecht as part of the current project, using open access fully automated techniques.[12] The best segmentation method was selected on a per cohort basis after visual inspection of the segmentation results. For six cohorts (i.e. Bundang VCI, CODECS, DEDEMAS, Hallym VCI, PROCRAS, and STROKDEM) the coroflo segmentation method was selected[34] whereas for COAST the bigrbrain segmentation method was selected.[35] For USCOG it was not possible to select a segmentation method on a per cohort basis due to heterogeneity of the imaging data and differences in quality of the different segmentation methods per patient. Therefore, selection of the segmentation method for USCOG was performed on a per subject basis. An expert (MC) with extensive experience on WMH segmentations visually inspected all segmentations. Scans with major disturbances due to technical issues such as scan quality or movement artefacts, or due to old infarcts or other pathology with apparent impact on WMH segmentations and thus volume estimates of an individual patient, were excluded. The acute infarct segmentations were subtracted from the WMH maps at a subsequent processing step. In total 93 (5.3%) of all segmentations failed and were excluded. For CASPER WMH segmentations were provided by the participating center, details are described elsewhere.[13]

Registration of WMH segmentations to the MNI-152 template

The registration of WMH segmentations to the MNI-152 brain template was performed centrally using RegLSM.[15] The FLAIR images were first registered to the corresponding T1 image with a linear registration. The T1 image was subsequently transformed to the T1 1-mm MNI-152 template, with a linear registration followed by a non-linear registration. An age-specific MRI template was used as an intermediate step before the final registration to MNI-152 space in order to improve the quality of the registration by providing a better match between patient and template.[14] The resulting transformations were combined into a single transformation that was subsequently used to transform the corresponding WMH map to the MNI-152 template. The final registration results of all cases were visually checked for accuracy and 89 patients (5.3%) with failed registrations were excluded.

To reduce heterogeneity and minimize the effects of possible misclassifications of other lesion types as WMH during the WMH segmentation procedures, voxels located outside the white matter (defined using the MNI probabilistic white matter atlas thresholded[14] at 30%) were removed from all individual WMH maps. As a final processing step, lesion maps of the acute infarct were subtracted from the WMH maps.

Lacune and old infarct ratings

Lacunes were identified visually, using STRIVE criteria.[16] In short, a lacune was defined as a round or ovoid, subcortical, fluid-filled cavity (signal similar to CSF) of between 3 mm and 15 mm in diameter. Lacunes were identified on the T1 sequence, with the FLAIR sequence and segmentations of the acute infarct used as reference in all cases. For visual identification of old infarcts (i.e. cortical and cerebellar infarcts and subcortical infarcts too large to meet criteria for lacunes), FLAIR and T1 sequences and the segmentation of the acute infarct were visualized simultaneously. Lesions were classified as old infarcts based on the following characteristics: 1)

gliosis (FLAIR), 2) volume loss due to infarction (T1) 3) damaged (cortical) tissue (both FLAIR/T1) 4) lesion not included in the yet available acute infarct segmentation 5) lesion did not meet the criteria of a lacune. All scans were screened for lacunes and old infarcts by two independent raters (FK and JMB or FK and GJB). In case of disagreement between ratings, consensus meetings were held. The scans of the CASPER cohort were already rated using the same criteria.

Atrophy measures
For all cohorts, the brain parenchymal fraction (BPF; total grey matter volume + total white matter volume divided by intracranial volume) was computed using the Computational Anatomy Toolbox (CAT) for SPM12.[36] For Hallym VCI and Bundang VCI, CAT for SPM12 did not generate reliable results, likely because of difficulty identifying the CSF/skull interface, therefore failing to measure ICV. FMRIB's Software Library (FSL)[37] was therefore used as an alternative method to compute BPF for these cohorts, but after visual inspection these segmentations did also not meet our quality control standards for the CSF spaces, including ventricles, and brain parenchyma. Consequently, Hallym VCI and Bundang VCI (n=1080) were excluded for the analyses that involved brain atrophy.

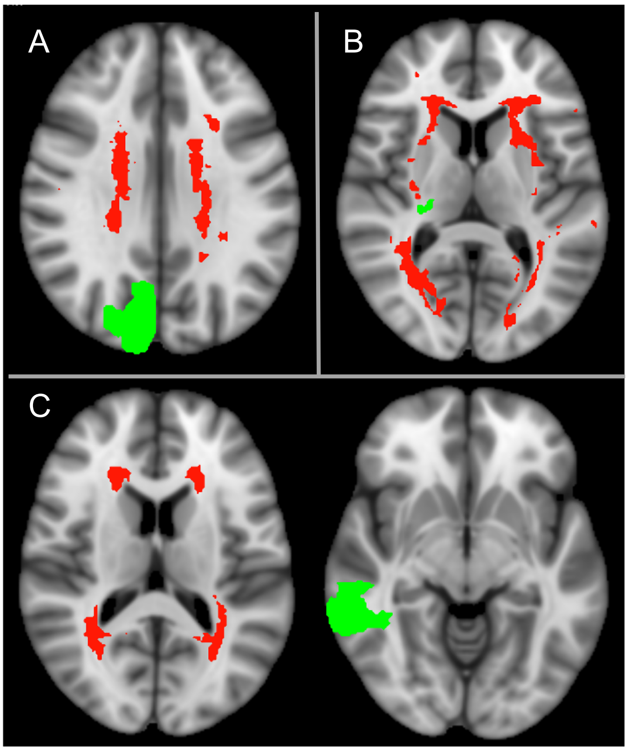

Figure S1. Examples of white matter hyperintensity and acute infarct lesion maps on the MNI-152 template

Panel A,B: Two examples of white matter hyperintensity lesion maps (in red) and corresponding acute infarct lesion maps (in green) at the same transversal slice. *Panel C:* Example of a white matter hyperintensity lesion map (in red) and the corresponding infarct lesion map (in green) at different slices. The infarcts shown at panel A and C are examples of the large infarct-type. The infarct shown at Panel B is an example of a small subcortical infarct-type.

Cognitive data processing and harmonization

Selection of neuropsychological tests

To reliably compare individual performance (z-scores) on cognitive domains, heterogeneity between cohorts was minimized by only selecting neuropsychological tests that were available in at least 40% of cohorts (selected tests were either truly identical or equivalent in both difficulty and cognitive construct measured). This process resulted in the selection of the following neuropsychological tests: TMT B, Digit Span Forward, Digit Span Backward, Phonemic Fluency (both 2 and 3 letter tests), Semantic Fluency (animal naming), TMT A, WAIS-R Digit Symbol Substitution Test (equivalent: Symbol Digit Modalities test) and the Boston Naming Test (equivalent: French D080 picture naming test). For verbal memory we included all word list recall tests measuring at least two of the following constructs: immediate recall, delayed recall and recognition (Rey Auditory Verbal Learning Test, Seoul Verbal Learning test, Word-List Recall, Free and Cued Selective Reminding Test and, the Word List Memory Task). Allocation of tests to specific cognitive domains was based on previous work.[7] Table S1 shows the selection of neuropsychological tests for each cohort.

Norm-referenced data

Cognitive performance at the level of individual neuropsychological tests was determined using local norms or normative data (corrected for age, educational level and sex where appropriate). Z-scores were calculated by the Utrecht team for the CODECS and USCOG cohorts, all other cohorts provided norm-referenced percentile scores or z-scores for each individual test. Percentile scores were converted to z-scores accordingly. Normative data for neuropsychological assessment per cohort are described in the supplements of prior work.[7] In addition, for DEDEMAS, the following norm-data were used: (1) z-scores of CERAD test battery[38] (including TMT B, Phonemic fluency, TMT A, Boston Naming Test, Semantic fluency-animals, Word-List Memory Task) were based on published norms using a standardized program (2) Z-scores of Digit Symbol Coding were calculated based on normative scores of Wechsler Adult Intelligence Scale, Third Edition (WAIS-III).[39]

Identification of outliers and construction of cognitive domain z-scores

Extreme scores were defined as the mean z-score of an individual test +/-3SD, on a per cohort basis. Extreme scores differed per test and per cohort and are probably the result of a combination of specific test characteristics (i.e. time-related tasks are more prone to generating extreme scores), patient characteristics (i.e. floor effects) and norm characteristics, that all differ per cohort. To reduce the impact of these (likely exaggerated) extreme z-scores, all extreme scores were set back to the cut-off value of the mean of the individual test +/-3SD for each individual cohort. The final z-scores of individual tests were used to calculate cognitive domain z-scores (mean of all available z-scores within one domain).

Table S1. Selection of cognitive tests per cohort

Cohort	Attention & Executive Functioning	Processing speed	Language	Verbal memory
Bundang VCI	1. TMT B<br>2. Phonemic fluency	1. TMT A<br>2. Digit Symbol Coding	1. Boston Naming Test<br>2. Semantic fluency –animals	Seoul Verbal Learning Test:<br>1. Immediate recall<br>2. Delayed recall<br>3. Recognition
CASPER	1. TMT B<br>2. Digit span forward<br>3. Digit span backward	1. TMT A	1. Semantic fluency – animals	Rey Auditory Verbal Learning Test<br>1. Immediate recall<br>2. Delayed recall<br>3. Recognition
COAST	1. Digit span forward<br>2. Digit span backward	1. Symbol Digit Modalities Test	1. Boston Naming Test<br>2. Semantic fluency – animals	Word-List Recall<br>1. Immediate<br>2. Delayed<br>3. Recognition
CODECS	1. TMT B<br>2. Phonemic fluency	1. TMT A	1. Semantic fluency –animals	N/A
DEDEMAS	1. TMT B<br>2. Phonemic fluency	1. TMT A<br>2. Digit Symbol Coding	1. Boston Naming Test<br>2. Semantic fluency – animals	Word-List Memory Task<br>1. Immediate<br>2. Delayed<br>3. Recognition
Hallym VCI	1. TMT B<br>2. Phonemic fluency	1. TMT A<br>2. Digit Symbol Coding	1. Boston Naming Test<br>2. Semantic fluency – animals	Seoul Verbal Learning Test<br>1. Immediate recall<br>2. Delayed recall<br>3. Recognition
PROCRAS	1. TMT B<br>2. Phonemic fluency<br>3. Digit span forward<br>4. Digit span backward	1. TMT A<br>2. Symbol Digit Modalities Test	1. Boston naming Test<br>2. Semantic fluency – animals	Rey Auditory Verbal Learning Test<br>1. Immediate recall<br>2. Delayed recall
STROKDEM	1. TMT B<br>2. Phonemic fluency	1. TMT A<br>2. Digit Symbol Coding	1. D080 – picture naming<br>2. Semantic fluency – animals	Free and Cued Selective Reminding Test<br>1. Immediate recall<br>2. Delayed free recall
USCOG	1. Phonemic fluency<br>2. Digit span forward<br>3. Digit span backward	N/A	1. Boston naming Test<br>2. Semantic fluency – animals	Rey Auditory Verbal Learning Test<br>1. Immediate recall<br>2. Delayed recall<br>3. Recognition

Abbreviations: N/A, not available; TMT, Trail Making Test.

Data quality control procedures
Standard operating procedures were followed to ensure that the fully processed lesion data matched the original imaging data and the clinical dataset provided by the participating center. These quality control procedures are complementary to procedures previously described by Weaver et al.[7] For each cohort, the Utrecht team selected a random subset of 10 subjects (n=2 for COAST) from the final merged database and checked the following:
1) If age, sex, education and a random sample of two cognitive test z-scores (before outliers were set back) were in line with received source data.
2) Cognitive harmonization steps: if mean domain z-scores were correctly calculated and whether outliers were set back where appropriate
3) If source imaging data and image processing logbooks were in line with final results of WMH registrations, presence of lacunes and old infarcts

Supplemental results

Table S2. Cohort-specific baseline characteristics

Cohort*	Bundang VCI (n = 546)	CASPER (n = 100)	COAST (n = 2)	CODECS (n = 11)	DEDEMAS (n = 66)	Hallym VCI (n = 534)	PROCRAS (n = 161)	STROKDEM (n = 135)	USCOG (n = 13)	Total sample (n = 1568)
**Demographics and clinical characteristics**										
Country of inclusion	South Korea	The Netherlands	Singapore	The Netherlands	Germany	South Korea	The Netherlands	France	The Netherlands	--
Age in years. mean (SD)	70.3 (10.7)	64.2 (10.6)	54.0 (9.9)	57.4 (15.3)	69.8 (8.6)	65.1 (11.9)	69.6 (9.5)	64.9 (12.0)	57.2 (17.7)	67.3 (11.5)
Sex, female. n (%)	324 (59.3)	26 (26.0)	1 (50)	5 (45.5)	24 (36.4)	232 (43.4)	57 (35.4)	83 (61.5)	7 (53.8)	626 (39.9)
Education level (STROKOG). n(%)†										
- Lower than secondary school	289 (52.9)	41 (41.0)	2 (100.0)	3 (27.3)	26 (39.4)	292 (54.7)	73 (45.3)	85 (63.0)	5 (38.5)	816 (52.0)
- Secondary school	106 (19.4)	18 (18.0)	0 (0)	2 (18.2)	23 (34.8)	126 (23.6)	49 (30.4)	15 (11.1)	4 (30.8)	343 (21.9)
- Technical school or college	26 (4.8)	33 (33.0)	0 (0)	3 (27.3)	16 (24.2)	27 (5.1)	34 (21.1)	11 (8.1)	3 (23.1)	153 (9.8)
- University or higher	125 (22.9)	8 (8.0)	0 (0)	3 (27.3)	1 (1.5)	89 (16.7)	5 (3.1)	24 (17.8)	1 (7.7)	256 (16.3)
NIHSS baseline. median (IQR)	3 (2-5)	N/A	9.5 (7-12)	0 (0-2)	2 (1-5)#	2 (1-4)||	3 (2-4.5)	0 (0-1)||	N/A	2 (1-4)||
IQCODE. median (IQR)	3.3 (3.1-3.7)||	3.1 (3.0-3.3)	3.1 (3.0-3.2)	N/A	N/A	3.1 (3-3.3)#	3.0 (3.0-3.1)	3.0 (3.0-3.1)	N/A	3.1 (3-3.4)#
History of stroke or TIA	79 (14.5)	6 (6.0)	0 (0)	0 (0)	8 (12.1)	74 (13.9)||	33 (20.5)	18 (13.3)	2 (15.4)	220 (14)§
**Brain imaging**										
Imaging timing. days‡. median (IQR)	5 (4-6)	87 (81-99)	3.5 (3-4)	97 (52-183)	4 (2.75-5)	1 (1-2)§	33 (27.5-40)	3 (3-4)	4 (3-6)	4 (2-7)§
WMH volume in mL. median (IQR)	14.6 (5.8-32.6)	4.9 (1.9-11.4)	0.1 (0.1-0.1)	2.4 (1.3-11.2)	2.8 (1.4-7.4)	6.9 (2.7-18.3)	4.8 (2.2-10.1)	2.7 (1.2-4.8)	3.8 (1.4-5.1)	7.1 (2.8-19.2)
Acute infarct volume in mL. median (IQR)	3.4 (1.1-14.3)	3.4 (0.9-13.2)	76.3 (46.2-106.4)	6.4 (1.1-21.8)	2.1 (0.5-12.6)	1.9 (0.9-8.3)	4.4 (1.4-21.1)	1.7 (0.6-8.7)	19.0 (2.4-66.2)	2.6 (1.0-12.3)

Table continues on next page

Stroke subgroup, n										
- Small subcortical infarcts	158	36	0	1	22	200	50	46	4	517
- Large infarcts	283	50	2	0	33	219	86	78	8	759
- Infratentorial infarcts	141	14	0	11	14	137	29	14	1	361
Presence of old infarcts. n (%)	101 (18.5)	21 (21.0)	1 (50.0)	2 (18.2)	10 (15.2)	103 (19.3)	27 (16.8)	37 (27.4)	0 (0)	302 (19.3)
Presence of lacunes. n (%)	216 (39.6)	30 (30.0)	1 (50.0)	5 (45.5)	28 (42.4)	181 (33.9)	70 (45.5)	38 (28.1)	3 (23.1)	572 (36.5)
**Cognitive assessment**										
Cognitive assessment timing. days‡. median (IQR)	103.5 (10-170)	87 (81-99)	106.8 (104-109.5)	90 (90-90)	187 (180-199)	98 (90-104)§	35 (28.5-40)	189 (178-201)	5 (3.5-12)	98 (72.5-151)§
Attention and executive functioning, z-scores, mean (SD)	-0.9 (1.1)#	-0.7 (0.7)	-0.4 (1.8)	-1.0 (0.8)	-0.1 (0.9)	-0.7 (1.1)||	-0.7 (0.8)§	-0.8 (1.6)||	-0.8 (0.6)	-0.7 (1.0)
Processing speed, z-scores, mean (SD)	-0.9 (1.2)#	-0.8 (1.3)	-2.6 (0.3)	-0.6 (0.7)	-0.1 (0.9)	-0.4 (1.1)||	-0.8 (1.1)§	-0.5 (1.1)||	N/A	-0.6 (1.2)
Language, z-scores, mean (SD)	-1.1 (1.2)§	-0.4 (0.8)#	-1.4 (1.5)	-0.6 (1.0)	0.0 (0.8)	-0.5 (0.9)	-1.0 (0.9)||	-0.2 (0.7)§	-1.0 (1.3)	-0.7 (1.1)
Verbal memory, z-scores, mean (SD)	-1.5 (1.1)||	0.0 (1.0)	-1.6 (1.0)	N/A	0.0 (0.8)	-0.7 (1.0)	-1.4 (0.9)	-0.2 (1.3)||	-1.4 (1.7)#	-0.9 (1.2).

*Cohort specific in- and exclusion criteria are described in Weaver et al (2021)[7], supplementary material p.20 and Weaver et al. (2019)[11], p.319. All cohort-specific references are listed elsewhere[7,11];†Education categories as defined by the STROKOG consortium[1]; ‡Days after index stroke; §Missing in <1%; ||Missing in 1-10%; #Missing in >10%, Abbreviations: SD, standard deviation; NIHSS, National Institute of Health Stroke Scale; IQR, interquartile range; TIA, transient ischemic attack; IQCODE, Informant Questionnaire for Cognitive Decline in the Elderly score; WMH, white matter hyperintensities.

Table S3. Baseline characteristics stratified by decile of white matter hyperintensity volume

Decile rank	1	2	3	4	5	6	7	8	9	10	Total sample
**Demographics**											
Age in years. mean (SD)	57.4 (11.3)	60.5 (11.9)	62.8 (10.8)	64.8 (11.8)	67.0 (11.0)	69.2 (9.5)	70.4 (8.8)	73.0 (8.1)	74.0 (7.6)	74.3 (9.1)	67.3 (11.5)
Sex. female. n (%)	52 (33.3)	62 (39.5)	59 (37.6)	50 (31.8)	62 (39.5)	62 (39.5)	55 (35.0)	54 (34.4)	71 (45.2)	99 (63.5)	626 (39.9)
NIHSS baseline. median (IQR)*	1 (0-3)	2 (1-4)	2 (1-4)	2 (1-4)	2 (1-5)	2 (1-4)	2 (1-5)	3 (1-4)	3 (1-5)	3 (1-5)	2 (1-4)
Infarct-type. n (%)											
Large infarcts	94	87	77	77	83	71	62	70	76	62	759
Small subcortical infarcts	43	44	53	51	43	59	63	48	52	61	517
Infratentorial infarcts	25	34	35	32	39	35	38	45	39	39	361
History of stroke and/or TIA*	9 (6.0)	18 (11.9)	15 (9.9)	23 (15.2)	18 (11.9)	22 (14.5)	28 (18.5)	28 (18.5)	25 (16.2)	31 (20.8)	217 (14.4)
Hypertension†	75 (48.1)	83 (52.9)	97 (61.8)	92 (58.6)	111 (70.7)	114 (72.6)	117 (74.5)	126 (80.3)	129 (82.2)	125 (80.1)	1069 (68.4)
Diabetes mellitus†	15 (9.6)	35 (22.3)	42 (26.8)	46 (29.3)	40 (25.5)	45 (28.7)	53 (33.8)	51 (32.5)	47 (29.9)	48 (30.8)	422 (27.0)
Smoking (past or present)‡	61 (50.8)	73 (58.9)	64 (48.1)	68 (51.9)	76 (51.7)	66 (45.8)	62 (42.8)	65 (44.2)	59 (38.1)	39 (26.0)	633 (45.3)
**Imaging parameters**											
WMH volume in mL. median (IQR)	0.7 (0.4-0.9)	1.7 (1.4-1.9)	2.8 (2.4-3.1)	4.2 (3.8-4.5)	5.9 (5.3-6.5)	8.6 (7.8-9.6)	12.5 (11.5-14.2)	19.2 (17.4-21.4)	33.0 (28.4-37.2)	58.3 (48.0-75.2)	7.1 (2.8-19.2)
Acute infarct volume in mL. median (IQR)	4.1 (0.9-21.2)	3.4 (0.8-19.6)	2.7 (0.9-10.9)	2.6 (1.0-13.4)	2.9 (0.9-13.0)	3.1 (1.1-13.2)	1.7 (0.9-12.5)	2.3 (0.9-10.5)	2.4 (1.1-11.1)	2.1 (1.0-6.4)	2.6 (1.0-12.3)
Presence of old infarct(s). n (%)	10 (6.4)	22 (14.0)	24 (15.3)	24 (15.3)	22 (14.0)	29 (18.5)	44 (28.0)	51 (32.5)	36 (22.9)	40 (25.6)	302 (19.3)
Presence of lacune(s). n (%)	15 (9.6)	19 (12.1)	33 (21.0)	58 (36.9)	46 (29.3)	53 (33.8)	75 (47.8)	75 (47.8)	88 (56.1)	110 (75.5)	572 (36.5)
Lacunes categories. n (%)											
0 lacunes	141 (90.4)	138 (87.9)	124 (79.0)	99 (63.1)	111 (70.7)	104 (66.2)	82 (52.2)	82 (52.5)	69 (43.9)	46 (29.5)	996 (63.5)
1 lacune	13 (8.3)	14 (8.9)	18 (11.5)	37 (23.6)	32 (20.4)	31 (19.7)	33 (21.0)	27 (17.2)	39 (24.8)	36 (23.1)	280 (17.9)
≥ 2 lacunes	2 (1.3)	5 (3.2)	15 (9.6)	21 (13.4)	14 (8.9)	22 (14.0)	42 (26.8)	48 (30.6)	49 (31.2)	74 (47.4)	292 (18.6)

*Missing in 1-10%; †Missing in <1%; ‡Missing in >10%; Abbreviations: IQR, interquartile range; SD, standard deviation; WMH, white matter hyperintensities; mL, milliliter; NIHSS, National Institute of Health Stroke Scale; TIA, transient ischemic attack.

Appendix S4. Sensitivity analysis on the role of atrophy using the brain parenchymal fraction

		Attention & Executive functioning*				Processing speed*				Language*				Verbal Memory*			
	Model	*n*	Coef	SE	P-value	*n*	Coef	SE	P-value	*n*	Coef	SE	P-value	*n*	Coef	SE	P-value
**Patients with BPF ratings (n=422)**	1a: WMH volume (univariate†)	*420*	-0.10	0.04	0.02	*406*	-0.22	0.06	<0.001	*393*	-0.05	0.05	0.3	*408*	-0.00	0.05	1.0
	1b: WMH volume (multivariate‡)		-0.08	0.05	0.09		-0.15	0.06	0.02		-0.06	0.05	0.3		-0.05	0.06	0.4
	1a + BPF		-0.10	0.05	0.03		-0.15	0.06	0.02		-0.03	0.05	0.5		0.02	0.06	0.7
	1b + BPF		-0.08	0.05	0.09		-0.14	0.06	0.02		-0.05	0.05	0.3		-0.04	0.06	0.5

*outcomes are mean z-scores; †log10-transformed, standardized; ‡fixed effects: age (standardized), sex (reference: female, category: male), education (reference: lower than secondary school, categories: secondary school, technical school/college completion, university or higher), acute infarct volume (log10-transformed, standardized), presence of lacune(s) (yes vs no), presence of old infarct(s) (yes vs no). All results were corrected for study site using random effects. A P-value of <0.05 was considered statistically significant. Abbreviations: BPF; brain parenchymal fraction; WMH, white matter hyperintensities; Coef, coefficient; SE, standard error.

This sensitivity analysis was done to assess the influence of BPF, as a indicator of brain atrophy, on the relation between WMH volume and domain-specific cognitive functioning. BPF was only available for a subset of 422 patients (27%) (see supplemental methods). Effect sizes of both the univariate and multivariate model largely remained unchanged after adding BPF to the model (model 1a versus model 1a + BPF and model 1b versus model 1b + BPF respectively). Groups are small and for the domains of language and VM the initial univariate coefficient for WMH volume is close to zero and non-significant.
The data thus suggest that the effect of atrophy on the relation between WMH volume and poststroke cognitive functioning in the domains of AEF and PS is limited. The role of atrophy in poststroke functioning on the domains of language and VM cannot be reliably assessed with the present data.

Appendix S5. Sensitivity analysis on the role of infarct location, using the location impact score

		Attention & Executive functioning*				Processing speed*				Language*				Verbal Memory*			
	Model	*n*	Coef	SE	P-value	*n*	Coef	SE	P-value	*n*	Coef	SE	P-value	*n*	Coef	SE	P-value
**All patients with location impact score (n=1502)**	WMH volume (univariate†)	*1404*	-0.21	0.03	<0.001	*1401*	-0.24	0.03	<0.001	*1471*	-0.18	0.03	<0.001	*1479*	-0.06	0.03	0.038
	WMH volume (multivariate‡)		-0.19	0.04	<0.001		-0.16	0.04	<0.001		-0.16	0.03	<0.001		-0.09	0.04	0.008
**Location impact score: 1st tertile (lowest; n=501)**	WMH volume (univariate)	*474*	-0.19	0.04	<0.001	*474*	-0.16	0.05	0.002	*488*	-0.20	0.04	<0.001	*488*	-0.02	0.05	0.6
	WMH volume (multivariate)		-0.09	0.05	0.09		-0.04	0.06	0.4		-0.16	0.05	0.001		-0.0	0.06	1.0
**Location impact score: 2nd tertile (middle; n=501)**	WMH volume (univariate)	*476*	-0.19	0.05	<0.001	*475*	-0.26	0.05	<0.001	*492*	-0.18	0.06	0.001	*500*	-0.03	0.06	0.5
	WMH volume (multivariate)		-0.22	0.06	<0.001		-0.23	0.07	<0.001		-0.18	0.07	0.008		-0.13	0.07	0.05
**Location impact score: 3rd tertile (highest; n=500)**	WMH volume (univariate)	*454*	-0.26	0.05	<0.001	*452*	-0.31	0.06	<0.001	*491*	-0.18	0.06	0.001	*491*	-0.03	0.06	0.5
	WMH volume (multivariate)		-0.24	0.07	<0.001		-0.24	0.07	0.001		-0.18	0.07	0.008		-0.13	0.07	0.05

*outcomes are mean z-scores; †log10-transformed, standardized; ‡fixed effects: age (standardized), sex (reference: female, category: male), education (reference: lower than secondary school, categories: secondary school, technical school/college completion, university or higher), geographic region (reference: Asia, category: Europe), acute infarct volume (log10-transformed, standardized), presence of lacune(s) (yes vs no), presence of old infarct(s) (yes vs no). All results were corrected for study site using random effects. A P-value of <0.05 was considered statistically significant. Abbreviations: WMH, white matter hyperintensities; Coef, coefficient; SE, standard error.

This sensitivity analysis was done to assess if having a strategic infarct (i.e. a lesion location with high risk of developing PSCI) would alter the relation (i.e. effect sizes) between WMH volume and poststroke cognitive functioning. Details on the location impact score, that provides risk estimates for the occurrence of PSCI according to infarct location, are described in prior work.[7] In short, using the acute infarct segmentations, voxel based lesion symptom mapping results (to relate infarct location to PSCI occurrence) were used to calculate this score for each individual participant: the location impact score is the mean coefficient (ie, ln[OR]) of voxels of the patient's acute infarct. Infarcts with high location impact scores are seen as strategic infarcts, the higher the score the greater risk of PSCI. For this analysis, all patients with availability of the location impact score from the Meta VCI Map strategic infarct location study[7] were included (n=1502). These patients were stratified into tertiles, based on their continuous location impact score. We did not use the original 5-point scale due to sample size constraints of subgroups. Stratified analyses were used because the risk of poor cognitive outcome (PSCI) is integrated in this score and it can therefore not be added as co-variate in the models. Results showed that the effect sizes for the relation between WMH volume and cognition was highest in the tertile with the highest location impact scores and lowest for the tertile with the lowest scores.
